# DT2216—a Bcl-xL-specific degrader is highly active against Bcl-xL-dependent T cell lymphomas

**DOI:** 10.1186/s13045-020-00928-9

**Published:** 2020-07-16

**Authors:** Yonghan He, Raphael Koch, Vivekananda Budamagunta, Peiyi Zhang, Xuan Zhang, Sajid Khan, Dinesh Thummuri, Yuma T. Ortiz, Xin Zhang, Dongwen Lv, Janet S. Wiegand, Wen Li, Adam C. Palmer, Guangrong Zheng, David M. Weinstock, Daohong Zhou

**Affiliations:** 1 Department of Pharmacodynamics, College of Pharmacy, University of Florida, Gainesville, FL USA; 2 Department of Hematology and Medical Oncology, University Medical Center Göttingen, Göttingen, Germany; 3 Department of Medicinal Chemistry, College of Pharmacy, University of Florida, Gainesville, FL USA; 4 Department of Pharmacology, School of Medicine, University of North Carolina, Chapel Hill, NC USA; 5 Department of Medical Oncology, Dana-Farber Cancer Institute and Harvard Medical School, 450 Brookline Avenue, Dana 510B, Boston, MA USA

**Keywords:** T cell lymphoma, Bcl-xL, VHL, PROTAC, Patient-derived xenograft

## Abstract

**Background:**

Patients with advanced T cell lymphomas (TCLs) have limited therapeutic options and poor outcomes in part because their TCLs evade apoptosis through upregulation of anti-apoptotic Bcl-2 proteins. Subsets of TCL cell lines, patient-derived xenografts (PDXs), and primary patient samples depend on Bcl-xL for survival. However, small molecule Bcl-xL inhibitors such as ABT263 have failed during clinical development due to on-target and dose-limiting thrombocytopenia.

**Methods:**

We have developed DT2216, a proteolysis targeting chimera (PROTAC) targeting Bcl-xL for degradation via Von Hippel-Lindau (VHL) E3 ligase, and shown that it has better anti-tumor activity but is less toxic to platelets compared to ABT263. Here, we examined the therapeutic potential of DT2216 for TCLs via testing its anti-TCL activity in vitro using MTS assay, immunoblotting, and flow cytometry and anti-TCL activity in vivo using TCL cell xenograft and PDX model in mice.

**Results:**

The results showed that DT2216 selectively killed various Bcl-xL-dependent TCL cells including MyLa cells in vitro. In vivo, DT2216 alone was highly effective against MyLa TCL xenografts in mice without causing significant thrombocytopenia or other toxicity. Furthermore, DT2216 combined with ABT199 (a selective Bcl-2 inhibitor) synergistically reduced disease burden and improved survival in a TCL PDX mouse model dependent on both Bcl-2 and Bcl-xL.

**Conclusions:**

These findings support the clinical testing of DT2216 in patients with Bcl-xL-dependent TCLs, both as a single agent and in rational combinations.

## Introduction

T cell lymphomas (TCLs) are a rare and heterogeneous group of lymphoid malignancies that account for approximately 10% of all non-Hodgkin lymphomas (NHLs) in high-income countries [1] and a higher fraction in lower- and middle-income countries [2]. About two thirds of TCLs are peripheral TCLs (PTCLs) and the remainder are cutaneous TCLs (CTCLs). The prognosis for patients with PTCLs and advanced-stage CTCLs are very poor because the majority of these TCL patients either fail to achieve a remission or experience relapse within 2 years after completion of first-line therapy [3]. Agents approved for relapsed/refractory TCL induce responses in a minority of patients with relapse-free survival typically less than 4 months [4, 5]. Therefore, new therapeutic agents that can more effectively treat TCL patients are urgently needed.

Like many other cancers, TCLs achieve resistance to therapy in part through overexpression of anti-apoptotic proteins in the Bcl-2 family, including Bcl-2, Bcl-xL, and Mcl-1 [6, 7]. Recently utilizing the BH3 profiling, a functional assay that directly interrogates the dependence on Bcl-2 anti-apoptotic proteins, we showed that nearly all CTCLs and a subset of PTCLs, including some patient-derived xenografts (PDXs) and primary patient samples, are dependent on Bcl-xL, which correlated with sensitivity to the Bcl-2/Bcl-xL inhibitor ABT263 (also known as navitoclax) but not the Bcl-2 inhibitor ABT199 (also known as venetoclax) [8]. These findings suggest that targeted inhibition of Bcl-xL could offer therapeutic benefits for some patients with TCL. Several Bcl-xL inhibitors, including the orally bioavailable ABT263, have been developed as new molecular targeted antitumor agents. However, these small molecule Bcl-xL inhibitors have failed during clinical development due to on-target and dose-limiting thrombocytopenia [9–11], as platelets are solely dependent on Bcl-xL for survival [9, 12].

We recently reported the development of DT2216 as a selective Bcl-xL degrader that has the potential to be developed as a safe antitumor agent because it spares platelets [13]. DT2216 targets Bcl-xL to the Von Hippel-Lindau (VHL) E3 ligase for degradation by the proteasome [13]. Because platelets express minimal levels of VHL, DT2216 can potently degrade Bcl-xL in various tumor cells but not in platelets, in a VHL- and proteasome-dependent manner. DT2216 was highly potent against various Bcl-xL-dependent leukemia and cancer cells because it effectively inhibited the growth of several xenograft tumors as a single agent (e.g., MOLT-4 T-ALL xenograft) or in combination with other therapeutic agents (e.g., H146 SCLC xenograft, MDA-MB231 breast cancer xenograft, and T-ALL PDX models) [13]. These previous findings suggest that DT2216 has great clinical potential than ABT263 or other Bcl-xL inhibitors. Before moving this promising agent to clinic, more cell and animal models are urgently needed to evaluate its efficacy and safety. However, the effect of DT2216 on TCL was unknown. Therefore, here we examined the therapeutic potential of DT2216 against different TCL cell lines in vitro and in TCL xenograft and PDX models. The results from our studies show that targeting Bcl-xL using DT2216 can selectively kill Bcl-xL-dependent TCL cells without causing significant platelet toxicity. Moreover, the combination of DT2216 with ABT199 may have broad therapeutic utility against TCLs that depend on both Bcl-xL and Bcl-2 for survival.

## Materials and methods

### Compounds

DT2216 and DT2216 negative compound (DT2216 NC) were synthesized in our lab according to the protocol as described previously [13]. ABT263 (Cat. No. S1001), ABT199 (Cat. No. S8048), S63845 (Cat. No. S8383), MG132 (Cat. No. S2619), doxorubicin (Cat. No. S1208), etoposide (Cat. No. S1225), and vincristine (Cat. No. S1241) were purchased from Selleckchem (Houston, TX, USA). A-1155463 (Cat. No. HY-19725) was purchased from MedChem Express (Monmouth Junction, NJ, USA).

### Cell culture

The TCL cell lines MyLa, MJ, MAC2A, L82, FEPD, SMZ1, and DL40 were cultured in RPMI medium supplemented with 10 or 20% FBS (Cat. No. 97068-085, VWR, Atlanta, GA, USA), and 1% penicillin-streptomycin (Cat. No. 15140122, Thermo Fisher Scientific) in a humidified incubator at 37 °C and 5% CO_2_. Specifically, MyLa cells were cultured in RPMI medium supplemented with 10% FBS and 100 units/mL IL-2 (PHC0021, Cat. No. 12430054, Thermo Fisher Scientific, Waltham, MA, USA). MJ and DL40 cells were cultured in RPMI medium supplemented with 20% FBS. MAC2A, L82, and SMZ1 cells were cultured in RPMI medium supplemented with 10% FBS. Human 786-O renal cell adenocarcinoma cells (Cat. No. CRL-1932) were purchased from American Type Culture Collection (ATCC, Manassas, VA, USA) and were cultured in DMEM (Cat. No. 12430054, Thermo Fisher Scientific, Waltham, MA, USA) supplemented with 10% FBS and 1% penicillin-streptomycin.

### Cell and platelet viability assay

Cells from different TCL cell lines were seeded into 96-well plates at 1 × 10^5^/well and treated for 72 h or indicated time points with various agents. Platelets were isolated from human platelet-rich plasma (PRP, Cat. No. SER-PRP-SDS, Zenbio, Research Triangle Park, NC, USA) [14]. Briefly, PRP was transferred into a 50-mL tube containing 5 mL acid citrate buffer (Cat. No. sc-214744, Santa Cruz Biotechnology, Dallas, TX, USA). To prevent clotting, prostaglandin E1 (PGE_1_, Cat. No. sc-201223A, Santa Cruz Biotechnology) and apyrase (Cat. No. A6237, Sigma-Aldrich) were added to final concentrations of 1 μM and 0.2 units/mL, respectively. After gently mixing the solution, platelets were pelleted by centrifugation at 1200×*g* for 10 min without a break. Pelleted platelets were gently washed in 2 mL HEPES Tyrode’s buffer (Cat. No. PY-921WB, Boston BioProducts, Ashland, MA, USA) containing 1 μM PGE_1_ and 0.2 units/mL apyrase. After washing, pellets were suspended in 10 mL HEPES Tyrode’s buffer containing 1 μM PGE_1_, 0.2 units/mL apyrase, and 10% FBS. Platelet number was counted using the HEMAVET 950FS hematology analyzer (Drew Scientific, Miami Lakes, FL, USA). For viability assays, platelet number was adjusted to 2 × 10^8^/mL in HEPES Tyrode’s buffer containing 1 μM PGE_1_, 0.2 units/mL apyrase and 10% FBS. Each treatment was performed in 2 mL platelet suspension in 15 mL polypropylene tubes. The tubes were placed on a rotating platform at room temperature, and the viability of platelets was measured after treatment for indicated time points. For measuring the viability, platelets were transferred to a 96-well plate (200 uL/well).

Cell and platelet viabilities were measured by the tetrazolium-based MTS assay according to the manufacturer’s instructions. Briefly, MTS reagent (2 mg/mL stock, Cat. No. G1111, Promega Madison, WI, USA) was freshly supplemented with phenazine methosulfate (PMS, 0.92 mg/mL stock, Cat. No. P9625, Sigma-Aldrich, St. Louis, MO, USA) at a 20:1 ratio, and 20 μL of this mixture was added to each control and treatment well. The cells and platelets were incubated for 4 h at 37 °C and 5% CO_2_, and then, the absorbance was recorded at 490 nm using Biotek’s Synergy Neo2 multimode plate reader (Biotek). The half maximal effective concentration (EC_50_) values of individual agents were calculated with the GraphPad Prism 7 software (GraphPad Software, La Jolla, CA, USA). The combination index (CI), EC_25_, EC_50_, and EC_75_ values were calculated using the Compusyn software **(**http://www.combosyn.com).

### Cell apoptosis assays

Cell apoptosis assay was done as described previously [15]. Briefly, cells were treated with vehicle or 10 μM Q-VD-OPh (QVD, Cat. No. S7311, Selleckchem, Houston, TX, USA) for 4 h prior to the addition of DT2216 for 24 h. Cells were harvested in polystyrene round-bottom tubes (Cat. No. 352058, Falcon, Corning, NY, USA). The cells were stained with Alexa Fluor 647-Annexin V (1:50, Cat. No. 640912, BioLegend, San Diego, CA, USA) and propidium iodide (PI, 10 μg/mL, Cat. No. 421301, BioLegend, San Diego, CA, USA) at room temperature for 30 min. Apoptotic cells were analyzed using flow cytometry (LSR II, BD Biosciences, San Jose, CA, USA).

### Immunoblotting

Proteins in cell lysates and tissue homogenates of the primary tumors from MyLa cell-engrafted mice or the spleens from DFTL-28776 cell-engrafted PDX mice were extracted using the RIPA buffer (Cat. No. BP-115DG, Boston BioProducts, Ashland, MA, USA) supplemented with 1% protease and phosphatase inhibitor cocktail (Cat. No. PPC1010, Sigma-Aldrich, St. Louis, MO, USA). Samples were lysed on ice for 30 min and then kept at − 80 °C freezer overnight. After centrifugation at 15,000×*g* at 4 °C for 15 min, the supernatant was collected and the protein concentration was measured using the Pierce BCA protein assay kit (Cat. No. 23225, Thermo Fisher Scientific). An equal amount of proteins (30–60 μg/lane) was loaded to a precast gel (Mini-PROTEAN TGX, Cat. No. 456-1094, Bio-Rad, Hercules, CA, USA) and transferred onto PVDF membranes (Invitrolon, Cat. No. LC2002, Life Technologies, Carlsbad, CA, USA) by electrophoresis. The membranes were blocked with 1X TBS-Tween (TBST, Cat. No. J77500, Affymetrix, Santan Clara, CA, USA) containing 5% non-fat dry milk (Cat. No. sc-2324, Santa Cruz Biotechnology, Dallas, TX, USA) and subsequently probed with primary antibodies at a predetermined optimal concentration overnight at 4 °C. The primary antibodies including Bcl-xL (Cat. No. 2762), Mcl-1 (Cat. No. 5453), Bcl-2 (Cat. No. 4223), Bax (Cat. No. 2772), Bak (Cat. No. 12105), Bim (Cat. No. 2933), Noxa (Cat. No. 14766), VHL (Cat. No. 68547), Caspase-3 (Cat. No. 9662), cleaved Caspase-3 (Cat. No. 9661), PARP (Cat. No. 9532), β-actin (Cat. No. 4970), and the secondary horse radish peroxidase (HRP)-linked antibody (Cat. No. 7074) were purchased from Cell Signaling Technology (Danvers, MA, USA). After washing with TBST for 3 times (10 min each time), the membranes were incubated with the secondary antibody for 2 h at room temperature. Following sufficient washing with TBST, the membranes were incubated with chemiluminescent HRP substrate (Cat. No. WBKLS0500, MilliporeSigma, Billerica, MA, USA). The blotting membranes were recorded using the ChemiDoc MP Imaging System (Bio-Rad, Hercules, CA, USA). The immunoblots were quantified using the ImageJ version 1.52a software.

### Quantitative polymerase chain reaction (qPCR)

Total RNA was extracted using an RNeasy Mini Kit (Cat. No. 74106, Qiagen, Gaithersburg, MD, USA). RNA (1 μg) was reverse-transcribed with the High-Capacity cDNA Reverse Transcription Kit (Cat. No. 4368813, Thermo Fisher Scientific). Real-time PCR was performed using specific TaqMan probes (Cat. No. 4351370, *BCL2L1* id: Hs00236329_m1; *BCL2* id: Hs00608023_m1 and *MCL1* id: Hs01050896_m1; *GAPDH* id: Hs02758991_g1) and the TaqMan Fast Advanced Master Mix (Cat. No. 4444965, Thermo Fisher Scientific). All reactions were run in triplicate on an ABI QuantStudio 3 Real-Time PCR System (Thermo Fisher Scientific). Data were normalized to *GAPDH* and calculated using the 2^– *∆∆CT*^ method [16].

### MyLa TCL xenograft mouse model and treatment

The animal work described in this study was approved by and done in accord with the Institutional Animal Care and Use Committee of the University of Florida. Female NOD-scid IL2Rg^null^ (NOD/SCID) mice were purchased from The Jackson Laboratory (Stock No. 005557; The Jackson Laboratory, Bar Harbor, ME, USA) at 5 weeks of age. They received food and water ad libitum and were allowed to acclimatize for 1 week before being used for experiments at the age of 6 weeks. One million (1 × 10^6^) MyLa cells were suspended in 100 μL of regular RPMI 1640 Medium (Cat. No. 22400089, Thermo Fisher Scientific, Waltham, MA, USA): Matrigel (Cat. No. 356231, Corning, New York, USA) 1:1 mixture and s.c. implanted in the right flank of NOD/SCID mice. Tumor growth was monitored daily and tumors were measured twice a week using Vernier caliper or digital calipers. Tumor volume was determined using the formula [(L × W^2^) × 0.5], where L is the length/long dimension in millimeter (mm) and W is the width/short dimension in millimeter. The treatment started once the average tumor volume reached 100 mm^3^. The animals were randomly assigned to treatment groups (*n* = 8 mice per group at the start of treatment) such that each group had a nearly equal starting average tumor volume. Mice were weighed twice a week, and the treatments were given according to average mouse weight within each group before initiation of treatment. They were treated with vehicle (10% ethanol, 30% PEG 400, and 60% PHOSAL 50 PG, once a day [qd] by gavage [p.o.]; 50% PHOSAL 50 PG, 45% MIGLYOL® 810 N, and 5% polysorbate 80, every 4 days [q4d] via intraperitoneal injection [i.p.]), ABT263 (formulated in 10% ethanol, 30% polyethylene glycol 400 (PEG 400), and 60% PHOSAL 50 PG; 50 mg/kg, qd/p.o.), or DT2216 (formulated in 50% PHOSAL 50 PG, 45% MIGLYOL® 810 N, and 5% polysorbate 80; 15 mg/kg, q4d/i.p.). PEG 400 (Cat. No. HR2-603) was from Hampton Research (Aliso Viejo, CA, USA). Ethanol (Cat. No. BP2818500) was from Fisher Scientific (Pittsburgh, PA, USA). PHOSAL 50 PG (Cat. No. 368315-3130003/020) was from American Lecithin Company (Oxford, CT, USA). MIGLYOL® 810 N was from IOI Oleochemical (Hamburg, Germany). Polysorbate 80 (Cat. No. P0138) was from Spectrum Chemical (New Brunswick, NJ, USA). Blood was collected 1 day after the first dose of the drugs for complete blood cell count (CBC) analysis. The mice were euthanized when the maximum tumor size reached the humane endpoint according to institutional policy concerning tumor endpoints in rodents. In addition, to prevent excessive pain or distress, the mice were euthanized if the tumors became ulcerated or the mice showed any signs of ill health. Mice were euthanized by CO_2_ suffocation followed by cervical dislocation, and various tissues including tumors were harvested for further analyses.

### DFTL-28776 T cell prolymphocytic leukemia (T-PLL) PDX model and treatment

Four-week-old female NOD/SCID mice from The Jackson Laboratory were allowed to acclimatize for 1 week before being used for experiments at the age of 5 weeks. DFTL-28776 cells were purchased from PRoXe (https://www.proxe.org/) and expanded once in NOD/SCID mice according to the instructions from PRoXe. They were injected into NOD/SCID mice via the tail veins at 1 × 10^6^ cells/mouse. Mice were weighed twice a week and DFTL-28776 cell engraftment in blood was monitored weekly by measuring human CD45^+^CD2^+^ cell engraftment in blood as described below. For the initial efficacy and survival study, DFTL-28776 cells were freshly harvested from the spleen of a host mouse with 60% of blood engraftment of DFTL-28776 cells and were immediately engrafted into NOD/SCID mice for further study. Mice with substantial engraftment of DFTL-28776 cells in blood (> 1.0%) were randomly assigned to different treatment groups (*n* = 5 mice for VEH and *n* = 4 mice for other groups at the start of treatment). They received treatment with vehicle, ABT199 (50 mg/kg, qd/p.o.), DT2216 (15 mg/kg, q4d/i.p.), or DT2216 plus ABT199. The mice were euthanized when they became moribund according to institutional policy concerning tumor endpoints in rodents by CO_2_ suffocation followed by cervical dislocation. The spleens were harvested from the mice for immunoblotting analyses of Bcl-xL, Bcl-2, and Mcl-1 expression. To assess additive vs. synergistic activity of DT2216 + ABT199 in DFTL-28776 TCL PDX mice, the Bliss independence model [17] was adapted to survival analysis as previously described [8]. A second study was performed to further characterize the effects of ABT199 and/or DT2216 on DFTL-28776 T-PLL PDX, in which previously frozen DFTL-28776 cells were used for the xenografts. Mice with a detectable engraftment of DFTL-28776 cells in blood (≥ 0.1%) were randomly assigned to different treatment groups (*n* = 6 mice per group); the average blood engraftment of DFTL-28776 cells per group was around 0.1%. They received treatment with vehicle, ABT263 (50 mg/kg, qd/p.o.), ABT199 (50 mg/kg, qd/p.o.), DT2216 (15 mg/kg, q4d/i.p.), or DT2216 plus ABT199. Vehicle-treated mice were euthanized 27 days after the initiation of the treatment due to their deteriorating condition, while the other groups were euthanized 7 days later. The spleen, liver, bone marrow, and blood were harvested. Age-matched female NOD/SCID control (CTL) mice (*n* = 5) without engraftment or any other treatments were included for comparative purposes. The weight of the spleens and livers from each mouse was measured and recorded. DFTL-28776 cell engraftment in the spleen, bone marrow, and blood were analyzed as described below.

### Analysis of DFTL-28776 cell engraftment in the spleen, bone marrow, and blood

Spleens were disaggregrated into RPMI 1640 medium supplemented with 2% FBS by grinding with plunger of a 3-mL sterile syringe (Cat. No. 309657, BD Biosciences, San Jose, CA, USA). The suspension was filtered through 70-μm cell strainers (Cat. No. 22-363-548, Thermo Fisher Scientific, Waltham, MA, USA). Bone marrow cells from the femora and tibiae were flushed into RPMI 1640 supplemented with 2% FBS using a 3-mL sterile syringe. Approximately 50 uL of blood was collected from each mouse via submandibular plexus route in an EDTA tube (Cat. No. 077051, RAM Scientific, Inc., Nashville, TN, USA). After red blood cells had been lysed with 1X BD Pharm Lyse™ lysing solution (Cat. No. 555899, BD Biosciences, San Jose, CA, USA), about 200,000 splenocytes, bone marrow cells, and white blood cells were stained with the mouse anti-human CD45 monoclonal antibody conjugated to BD Horizon™ V450 (Cat. No. 560367; BD Biosciences, San Jose, CA, USA) and mouse anti-human CD2 monoclonal antibody conjugated to APC (Cat. No. 300214; BioLegend, San Diego, CA, USA). Human CD45^+^CD2^+^ cells were analyzed on an Aurora flow cytometer (Cytek Aurora, Fremont, CA, USA).

### DFTL-28776 PDX cell purification and analyses

When DFTL-28776 cell engraftment in blood reached approximately 60%, DFTL-28776 PDX mice were euthanized by CO_2_ suffocation followed by cervical dislocation. The spleens were harvested from the mice for the preparation of single splenocyte suspension. DFTL-28776 cells were isolated using the EasySep Mouse/Human Chimera Isolation Kit (Cat. No. 19849; StemCell Technologies, Vancouver, BC, Canada) according to the manufacturer’s instructions. Cell viability of isolated DFTL-28776 cells after treatment with different agents was measured by tetrazolium-based MTS assay as described above with a slight modification. Briefly, DFTL-28776 cells were seeded into 96-well plates at 2 × 10^5^/well and treated for 24 h with various agents, the absorbance was read after adding the MTS reagents and incubation with cells overnight. All other assays for DFTL-28776 cells were done in a way similar to the assays for other TCL cell lines described above.

### Hematoxylin & eosin staining and anti-hCD45 immunostaining of the spleens and livers of DFTL-28776 PDX mice

The formalin-fixed tissues of mice from different groups were embedded in paraffin, sectioned into 5-μM slices, and mounted onto glass slides. Hematoxylin & eosin (HE) staining was done by the Molecular Pathology Core of the University of Florida. A set of slides was used for immunostaining of human CD45 (Cat. No. M0701, DAKO, Agilent Technologies, Inc., Santa Clara, CA, USA). Slides were immersed twice (5 min each time) in xylene solution followed by serial washes with 100, 95, and 75% ethanol, respectively (2 min each time). Then, the slides were dipped in water twice and then incubated in trilogy solution (Cat. No. 920P-10, Cell Marque Corporation, Rocklin, CA, USA) at 95 °C for 25 min. After this incubation, the slides were washed under running water for 1 min and then washed with 1X TBST for 5 min. After incubation in 1.5% horse serum (Cat. No. ZE0122, Vector Laboratories, Inc., Burlingame, CA, USA) for 30 min, the slides were washed with TBST again and then sequentially incubated with avidin and biotin blocking solutions in a commercial kit (Cat. No. ZE0919, Vector Laboratories, Inc., Burlingame, CA, USA) following the manufacturer’s instructions. The slides were washed with TBST and incubated with mouse monoclonal anti-human CD45 primary antibody (1:50) overnight followed by three washes with TBST. Slides were incubated with anti-mouse secondary antibody (1:200) for 30 min, washed again with TBST, followed by incubation with reagents in the Vectastain Alkaline Phosphatase Standard AK-5100 kit (Cat. No. ZE0628, Vector Laboratories, Inc., Burlingame, CA, USA). Finally, the slides were washed with TBST and mounted with a coverslip using the mounting medium with DAPI (Cat. No. ZD0421C, Vector Laboratories, Inc., Burlingame, CA, USA). The slides were visualized under a fluorescent microscope (Olympus BX43 with Olympus DP80 camera) at a × 200 magnification to obtain images.

### Complete blood cell counts

Approximately 50 μL of blood was collected from each mouse in EDTA tubes via the submandibular plexus. The blood was immediately used for CBC analysis using the HEMAVET 950FS hematology analyzer (Drew Scientific, Miami Lakes, FL, USA). The data were expressed as the number of different blood cells or platelets per microliter of blood.

### Statistical analysis

Most of the graphs presented in this manuscript were made and statistical analyses were performed using GraphPad Prism 7 software (GraphPad Software, San Diego, CA, USA). For analysis of means of three or more groups, analysis of variance (ANOVA) tests were performed. In the event that ANOVA justified post hoc comparisons between group means, the comparisons were conducted using Tukey’s multiple comparison test. Two-sided unpaired Student’s *t* test was used for comparisons between the means of two groups. Kaplan-Meier test was used for the survival rate analysis, and the data were statistically analyzed using log-rank (Mantel-Cox) test. The Bliss independence model was adapted to assess additive vs. synergistic activity of DT2216 + ABT199 in the DFTL-28776 TCL PDX mice as we previously described [8]. *p* < 0.05 was considered statistically significant.

## Results

### DT2216 selectively kills Bcl-xL-dependent TCL cell lines by degrading Bcl-xL in a VHL-dependent manner

**Fig. 1:**
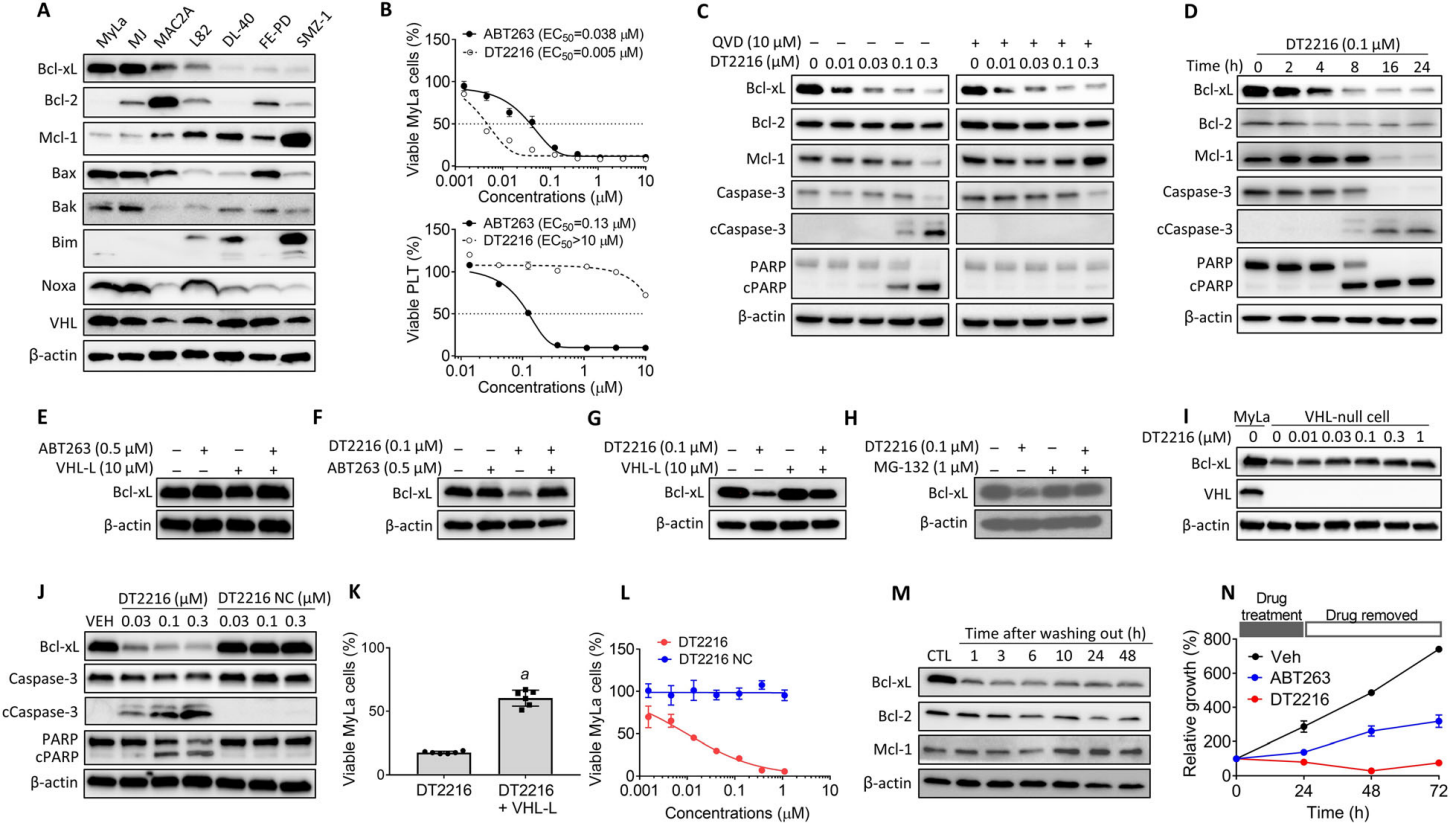
DT2216 selectively kills Bcl-xL-dependent TCL cell lines by degrading Bcl-xL in a VHL-dependent manner. **a** A represent immunoblot analysis of expression of various Bcl-2 family proteins and VHL in selective TCL cell lines. **b** Effects of DT2216 and ABT263 on MyLa cell and platelet (PLT) viability after 72 h treatment. The data are mean ± SEM (*n* = 3 independent assays). **c** DT2216 induced Bcl-xL degradation in a dose-dependent manner. MyLa cells were pretreated with or without QVD for 4 h, and then treated with DT2216 for 16 h. **d** DT2216 induced Bcl-xL degradation in a time-dependent manner. **e** ABT263 and/or the VHL ligand (VHL-L) could not induce Bcl-xL degradation. **f** ABT263 pretreatment blocked the degradation of Bcl-xL by DT2216. **g** VHL-L pretreatment blocked the degradation of Bcl-xL by DT2216. **h** MG132 blocked the degradation of Bcl-xL by DT2216. **i** DT2216 could not induce Bcl-xL degradation in VHL-null 786-O cells. **j** DT2216, but not its negative control compound (DT2216 NC), induced Bcl-xL degradation and cleavage of caspase-3 and PARP. For Fig. 1e–h, MyLa cells were pretreated with indicated compounds for 2 h, and proteins were measured 16 h after treatment with DT2216. **k** Pretreatment with VHL-L blocked the effect of DT2216 on cell viability. MyLa cells were pretreated with VHL-L for 2 h, and then treated with DT2216 for 72 h. The data are mean ± SD (*n* = 6 replicates). a *p* < 0.05 vs. DT2216. **l** DT2216, but not DT2216 NC, reduced the cell viability that was assayed after treatment with DT2216 or DT2216 NC for 72 h. The data are mean ± SD (*n* = 6 replicates). **m** DT2216-induced Bcl-xL degradation persisted up to 48 h upon the removal of DT2216 after 24 h DT2216 treatment. **n** The effect of DT2216, but not ABT263, on reducing MyLa cell viability, persisted up to 48 h upon the removal of ABT263 or DT2216 after 24 h treatment. The data presented are mean ± SD (*n* = 3 replicates)

Using the BH3 profiling assay, our previous studies revealed that a fraction of human TCL cell lines are exclusively dependent on Bcl-xL for survival and are highly sensitive to Bcl-xL-specific and dual Bcl-2/Bcl-xL inhibitors [8]. We tested four Bcl-xL-dependent TCL cell lines (i.e., MyLa, MJ, MAC2A, and L82), and all of them expressed high levels of Bcl-xL and low levels of Bim (Fig. 1a) and were relatively resistant to conventional chemotherapy drugs, but were highly sensitive to DT2216 (EC_50_, 5–280 nM) (Fig. 1b and Table 1). In contrast, TCL lines (DL40, SMZ1, and FEPD) found to depend on Mcl-1 for survival by BH3 profiling [8] expressed relatively higher levels of Mcl-1 and were resistant to DT2216 (EC_50_ > 10,000 nM) (Fig. 1a and Table 1). Notably, the three lines resistant to DT2216 lacked expression of Bcl-xL (Fig. 1a). All 7 cell lines expressed variable levels of Bax, Bak, and Noxa and similar levels of VHL (Fig. 1a). As we previously showed [13], human platelets were highly sensitive to ABT263 but very resistant to DT2216 compared with Bcl-xL-dependent TCL cells because platelets express minimal levels of VHL to degrade Bcl-xL (Fig. 1b, Table 1, and Supplementary Fig. S1A, B).

**Table 1:** EC_50_ of DT2216, Bcl-2 family protein inhibitors, or chemotherapy drugs against selective T cell lymphoma (TCL) cell lines

TCL cell lines	Chemotherapy drugs (nM)			Bcl-2 family inhibitors (μM)				DT2216 (μM)
	Doxorubicin	Etoposide	Vincristine	ABT263	A-1155463	ABT199	S63845	(a selective Bcl-xL
				(Bcl-2/xL)	(Bcl-xL)	(Bcl-2)	(Mcl-1)	degrader)
MyLa (CTCL)	124.60	199.50	8.60	0.04	0.0008	> 5	> 5	0.005
MJ (CTCL)	480	> 10,000	60	0.33	0.30	> 10	> 10	0.10
MAC2A (ALK^-^ALCL)	120	1260	< 1	0.06	0.009	1.80	> 10	0.28
L82 (ALK^+^ALCL)	30	110	< 1	0.07	0.03	2.38	1.55	0.02
DL40 (ALK^-^ALCL)	10	80	< 1	4.42	> 10	> 10	0.003	> 10
SMZ1 (PTCL-NOS)	10	100	1.50	9.71	> 10	> 10	0.05	> 10
FEPD (ALK^-^ALCL)	60	370	10	7.90	> 10	8.99	0.22	> 10
**Human PLT**	–	–	–	0.13	–	–	–	> 10

The results were calculated from a representative experiment with 6 replicates per concentration for each compound. Similar results were also got in two or more assays

*EC*_*50*_ the half maximal effective concentration, *CTCL* cutaneous TCL, *ALK*^*-*^*ALCL* anaplastic lymphoma kinase negative anaplastic large cell lymphoma, *ALK*^*+*^*ALCL* anaplastic lymphoma kinase positive anaplastic large cell lymphoma, *PTCL-NOS* peripheral TCL not otherwise specified, *PLT* platelets

To validate the mechanism of action of DT2216 against TCL cells, we used MyLa cells as a model system as they are solely dependent on Bcl-xL for survival and are highly sensitive to DT2216 (Table 1). We first examined if DT2216 kills MyLa cells via degrading Bcl-xL and inducing apoptosis. We found that DT2216 dose- and time-dependently decreased the expression of Bcl-xL but had no effect on the expression of *BCL2L1* (the gene that encodes Bcl-xL) mRNA in MyLa cells (Fig. 1c, d and Supplementary Fig. S1C–E). The decrease in Bcl-xL expression was associated with the activation of caspase-3, cleavage of poly ADP ribose polymerase (PARP), and induction of apoptosis, all of which were abrogated by pretreating the cells with QVD-OPh (QVD, a pan-caspase inhibitor) (Fig. 1c, d and Supplementary Fig. S1F). Although at higher concentrations (> 0.1 μM) DT2216 also reduced the levels of Mcl-1, this effect was likely attributable to the activation of caspase-3 resulting from DT2216-induced Bcl-xL degradation. This suggestion is supported by the observation that inhibition of caspase activity with QVD abrogates the effect of DT2216 on Mcl-1 (Fig. 1c). Mcl-1 is a known caspase-3 substrate [18], and we previously showed that DT2216 does not bind to Mcl-1 and cannot degrade Mcl-1 directly [13].

Next, we tested whether DT2216 degrades Bcl-xL in a VHL- and proteasome-dependent manner in TCL cells. First, we examined the effects of ABT263 and VHL ligand (VHL-L) alone and in combination on Bcl-xL levels in MyLa cells and found that neither affected the level of Bcl-xL (Fig. 1e). In addition, we found that pre-incubation of MyLa cells with an excess amount of ABT263 or VHL-L blocked the degradation of Bcl-xL induced by DT2216 (Fig. 1f, g). Addition of the proteasome inhibitor MG-132 had the same effect (Fig. 1h). DT2216 had no effect on the levels of Bcl-xL in VHL-null cells (Fig. 1i). A negative control molecule, DT2216 NC, which binds Bcl-xL but does not bind VHL [13] was incapable of reducing the level of Bcl-xL and activating caspase-3 in MyLa cells (Fig. 1j). Moreover, the effect of DT2216 on MyLa cell viability was also dependent on its proteolysis targeting chimera (PROTAC) activity because pre-incubation of MyLa cells with an excess amount of VHL-L not only inhibited DT2216-induced Bcl-xL degradation but also reduced the cytotoxicity of DT2216 (Fig. 1g, k). Finally, DT2216 NC showed no cytotoxicity against MyLa cells (Fig. 1l). Similar results were observed in other Bcl-xL-dependent TCL cell lines (Supplementary Fig. S2). Collectively, these data confirm that DT2216 acts as a PROTAC that depends on the VHL E3 ligase and proteasome to degrade Bcl-xL and induce apoptosis in Bcl-xL-dependent TCL cells.

Because DT2216 functions as a Bcl-xL PROTAC, it can induce protein degradation and then be recycled and thereby function in a sub-stoichiometric manner. Unlike traditional occupancy-driven protein Bcl-xL inhibitors such as ABT263, its effects should not be limited by equilibrium occupancy [19–23]. Therefore, DT2216 should be more potent and have longer-acting activity after washout than ABT263 [24–30]. This hypothesis is supported by the finding that DT2216 reduced the levels of Bcl-xL in MyLa cells even after DT2216 was removed from the culture for up to 48 h compared to cells without DT2216 treatment (Fig. 1m). Furthermore, MyLa cells resumed proliferation within 24 h after the removal of ABT263 in the culture, whereas the proliferation of MyLa cells remained to be suppressed even 48 h after the removal of DT2216 (Fig. 1n).

### DT2216 is more potent against TCL but less toxic to platelets than ABT263 in vivo

**Fig. 2:**
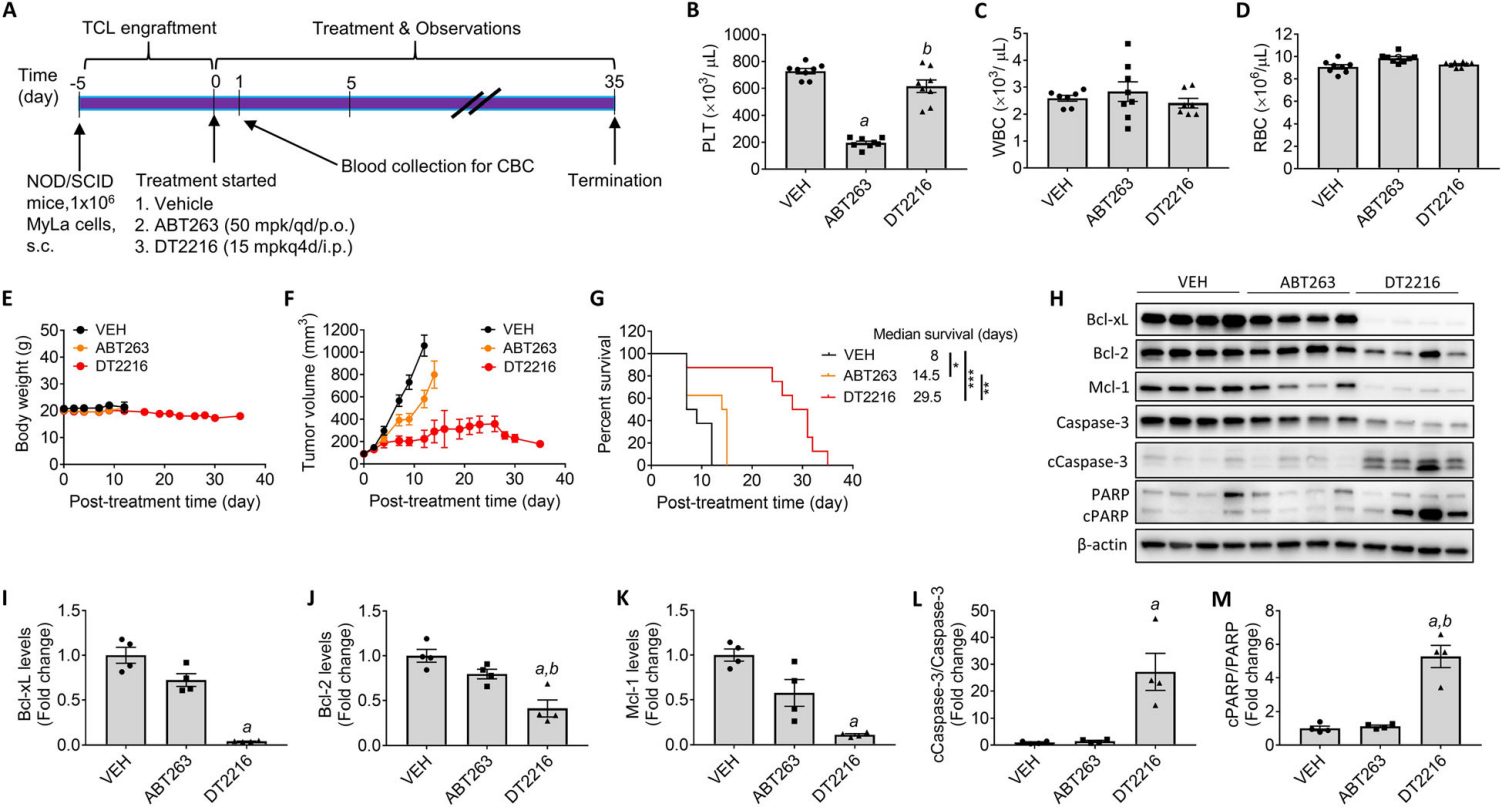
DT2216 is more potent against TCL but less toxic to platelets than ABT263 in vivo. **a** Illustration of the experimental design of the MyLa xenograft model. **b**–**d** Blood platelet (PLT), white blood cell (WBC), and red blood cell (RBC) counts in mice were measured 1 day after receiving the first dose of vehicle (VEH), ABT263, or DT2216. The data presented are mean ± SEM (*n* = 8 mice per group at the start of treatment). a and b, *p* < 0.05 vs. VEH and ABT263, respectively. **e** Body weight changes in MyLa tumor-bearing mice after the start of treatment with VEH, ABT263, or DT2216 as shown in **a**. Data are presented as mean ± SEM (*n* = 8 mice per group at the start of treatment). **f** Changes in tumor volume over time after the start of treatment as shown in **a**. Data are presented as mean ± SEM (*n* = 8 mice per group at the start of treatment). **g** Kaplan–Meier survival curve showing the survival of mice after MyLa engraftment. Median survival time within each treatment group is presented along with statistical analysis results (*n* = 8 mice in each group at the start of treatment). *p < 0.05, **p < 0.01, and ***p < 0.001 in indicated comparisons. **h** Immunoblot analysis of Bcl-xL, Bcl-2, Mcl-1, Caspase-3, and PARP in primary tumors (*n* = 4 mice in each group). Proteins were extracted from the primary tumors after euthanization when mice reached the experimental endpoint as described in the “Materials and methods” section. **i**–**m** Quantification of Bcl-xL, Bcl-2, Mcl-1, Caspase-3, and PARP as shown in **h**. a and b, *p* < 0.05 vs. VEH and ABT263, respectively. Data are presented as mean ± SEM (*n* = 4 mice per group). cCaspase-3, cleaved or activated caspase-3; PARP, poly ADP ribose polymerase; cPARP, cleaved PARP

Next, we compared DT2216 to ABT263 in vivo by xenografting MyLa cells s.c. into immunodeficient mice. MyLa-engrafted mice were randomized into vehicle, ABT263 (50 mpk/qd/p.o.), or DT2216 (15 mpk/q4d/i.p.) treatment groups when their tumors reached ~ 100 mm^3^ (Fig. 2a). The dose of ABT263 used for this experiment was selected to avoid the ABT263 treatment-induced severe thrombocytopenia we observed in our recent study [13]. One day after the first treatment, the blood was collected from each mouse for a CBC analysis. ABT263-treated mice had ~ 73% reduction in blood platelet counts compared to vehicle-treated mice, whereas DT2216-treated mice had ~ 16% reduction (Fig. 2b). Neither treatment significantly affected other blood cell counts (Fig. 2c, d). Mice were continued on treatment until they became moribund or their tumors were close to 1000 mm^3^ or ulcerated. Neither ABT263 nor DT2216 significantly affected body weight (Fig. 2e). ABT263 treatment slightly slowed the tumor growth and moderately extended the median survival time of the mice (from 8 to 14.5 days) compared to vehicle treatment (Fig. 2f, g). In contrast, DT2216 treatment markedly suppressed the primary tumor growth and extended the median survival time of the mice to 29.5 days (Fig. 2f, g). More importantly, the marked tumor suppression induced by DT2216 was associated with a more than 95% reduction in Bcl-xL levels, substantial decreases in Bcl-2 and Mcl-1 levels, and significant activation of caspase-3 and cleavage of PARP in the tumors collected from the mice (Fig. 2h–m). The decreases in tumor expression of Bcl-2 and Mcl-1 after DT2216 treatment were likely due to the activation of caspase-3 and induction of MyLa cell apoptosis (as shown in Fig. 1c) [18]. In contrast, ABT263 treatment had no significant effect on the tumor expression of these Bcl-2 family proteins, nor did it induce significant activation of caspase-3 and cleavage of PARP in the tumors (Fig. 2h–m). These findings demonstrate that DT2216 is more potent against MyLa TCL cells but less toxic to platelets than ABT263 in mice.

### DT2216 can synergistically kill TCL PDX cells in combination with ABT199 in vitro

**Fig. 3:**
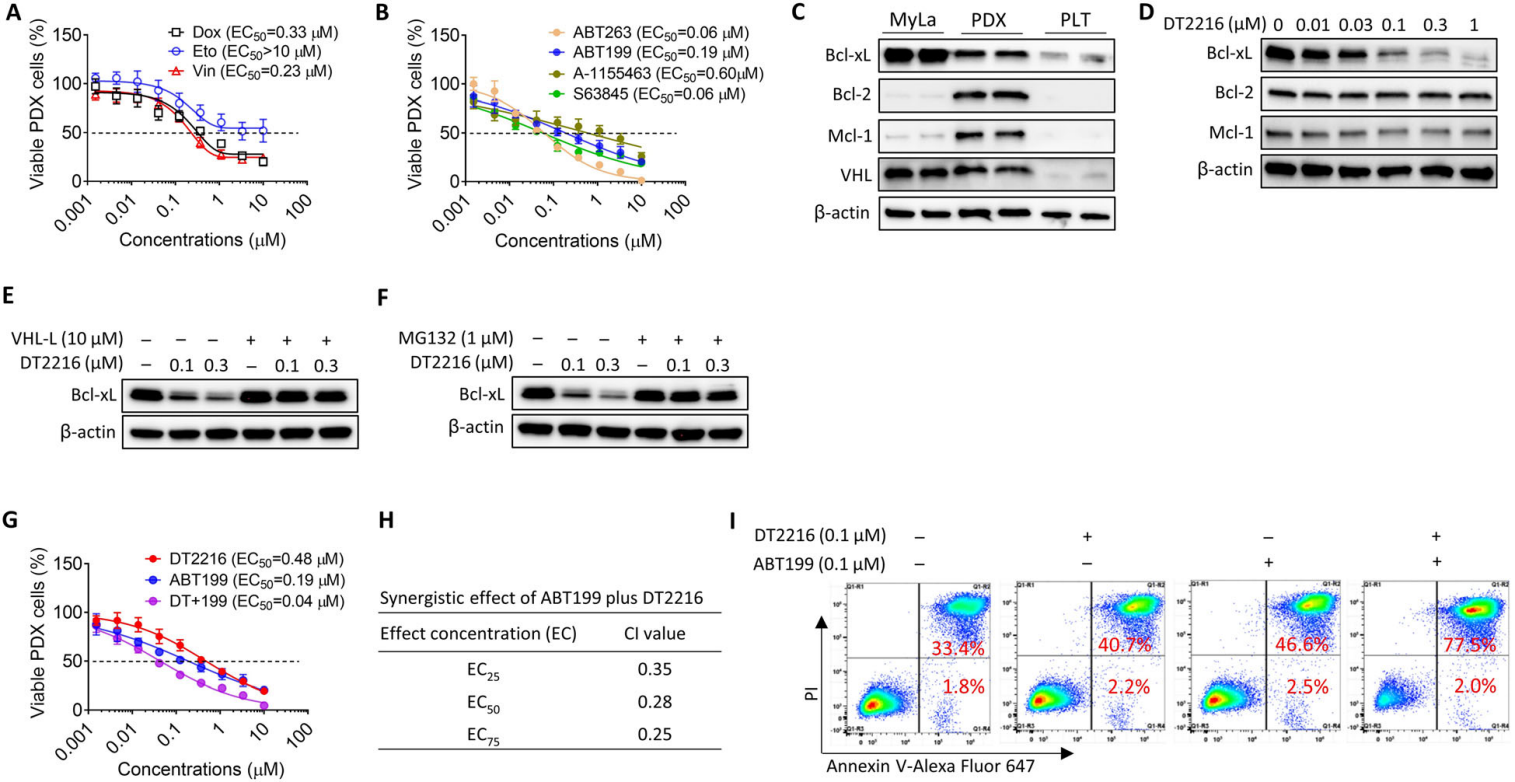
DT2216 can synergistically kill TCL PDX cells in combination with ABT199 in vitro*.***a** The viability of DFTL-28776 PDX cells was determined 24 h after doxorubicin (Dox), etoposide (Eto), or vincristine (Vin) treatment. EC_50_, half maximal effective concentration. The data presented are mean ± SD (*n* = 2 independent assays, with 3 replicates in each assay). **b** The viability of DFTL-28776 PDX cells was determined 24 h after ABT263, ABT199, A-1155463, or S63845 treatment. The data presented are mean ± SD (*n* = 2 independent assays, with 3 replicates in each assay). **c** Bcl-xL, Bcl-2, Mcl-1, and VHL expressions in MyLa cells, DFTL-28776 PDX cells, and PLT. **d** DT2216 degraded Bcl-xL in DFTL-28776 PDX cells after treatment with indicated concentrations of DT2216 for 16 h. **e** VHL ligand (VHL-L) pretreatment blocked the degradation of Bcl-xL induced by DT2216 in DFTL-28776 PDX cells. The PDX cells were pretreated with 10 μM VHL-L for 2 h, and then treated with indicated concentrations of DT2216 for 16 h. **f** Proteasome inhibition with MG132 blocked the degradation of Bcl-xL induced by DT2216 in DFTL-28776 PDX cells. The PDX cells were pretreated with 1 μM MG-132 for 2 h and then treated with the indicated concentrations of DT2216 for 16 h. **g** The viability of DFTL-28776 PDX cells was determined 24 h after treatment with DT2216, ABT199, or DT2216 plus ABT199 (DT+199 at a ratio of 1:1). The data presented are mean ± SD (*n* = 2 independent assays, with 3 replicates in each assay). **h** The combination index (CI) values of DT+199 group at EC_25_, EC_50_, and EC_75_, values calculated from the data presented in **g** are presented. **i** Combination of DT2216 with ABT199 induced apoptosis in PDX cells. Apoptosis was assayed after treatment with 0.1 μM DT2216, 0.1 μM ABT199, or combination of 0.1 μM DT2216 with 0.1 μM ABT199 for 24 h. PI, propidium iodide

We previously established a panel of TCL PDX models and demonstrated that these TCL PDX models capture various aspects of TCL biology and found that multiple TCL models co-depend on more than one Bcl-2 family member [8, 31]. We showed that a PDX of T cell prolymphocytic leukemia (T-PLL; DFTL-28776) is co-dependent on Bcl-xL and Bcl-2 [8]. In fact, treatment of DFTL-28776 PDX mice with the Mcl-1 inhibitor AZD5991 failed to induce any benefit compared to vehicle [8]. T-PLL is a rare but aggressive disease that involves the peripheral blood, bone marrow, lymph nodes, liver, and spleen. T-PLL is considered refractory to conventional chemotherapy and requires allogeneic stem cell transplantation for cure [32]. As expected, DFTL-28776 cells were highly resistant to doxorubicin, etoposide, and vincristine (Fig. 3a and Supplementary Table S1). They were also relatively resistant to ABT199, A-155463 (a selective Bcl-xL inhibitor), and S63845 (a selective Mcl-1 inhibitor) (Fig. 3b and Supplementary Table S1). However, ABT263 potently induced cell death, confirming dependence on both Bcl-xL and Bcl-2 for survival (Fig. 3b and Supplementary Table S1). Of note, DFTL-28776 cells express high levels of Bcl-xL, Bcl-2, and Mcl-1 (Fig. 3c), indicating that protein levels are not predictive of response.

DT2216 induced Bcl-xL degradation in DFTL-28776 cells in vitro in a dose-dependent fashion (Fig. 3d). Both VHL-L and MG-132 blocked Bcl-xL degradation induced by DT2216, consistent with the effects being dependent on VHL and the proteasome (Fig. 3e, f). Because DFTL-28776 cells depend on both Bcl-2 and Bcl-xL, we examined whether DT2216 could more effectively kill the cells in combination with ABT199. The combination of DT2216 and ABT199 (ratio 1:1) synergistically killed DFTL-28776 cells in vitro via induction of apoptosis (Fig. 3g–i) and their combined effects were similar to ABT263 (Fig. 3b, g).

### DT2216 is more effective against T-PLL PDX cells when combined with ABT199 in vivo

**Fig. 4:**
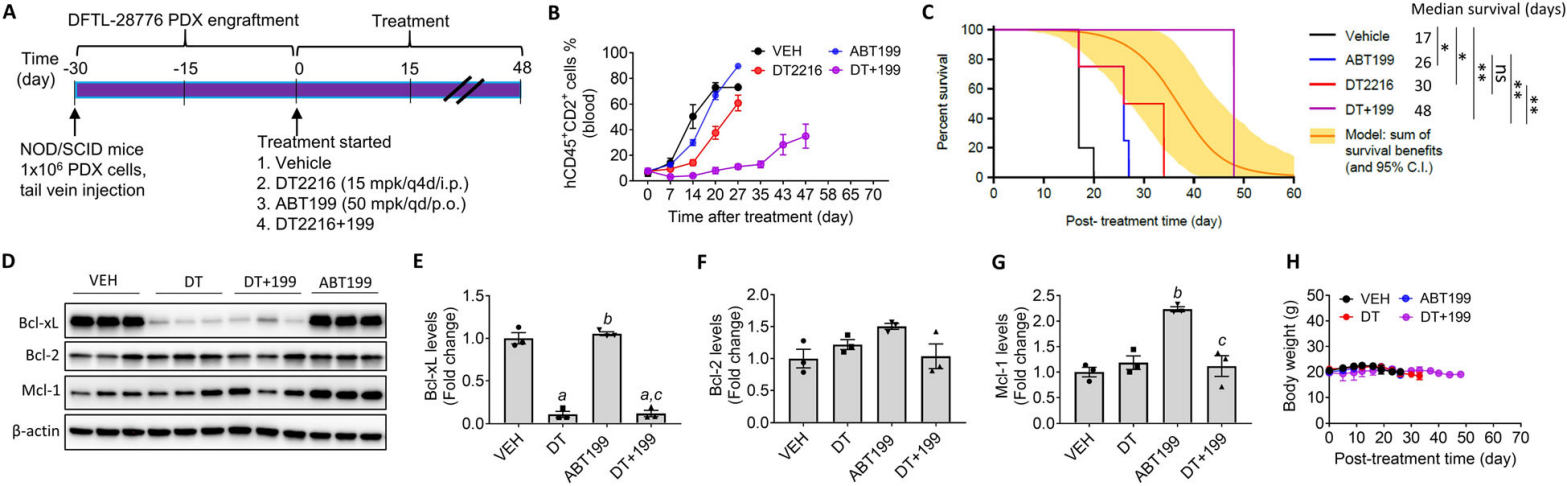
DT2216 combined with ABT199 synergistically reduced blood engraftment and improved survival in a PDX model*.***a** Illustration of the experimental design of the first study on DFTL-28776 PDX xenograft model. **b** Changes in blood DFTL-28776 PDX cell burden over time after the start of treatment as shown in **a**. Data are presented as mean ± SEM (*n* = 5 for VEH and *n* = 4 for other groups at the start of treatment). **c** Survival curve showing the survival of mice after the TCL PDX cell engraftment. Median survival time within each treatment group is presented along with statistical analysis results (*n* = 5 for VEH and *n* = 4 for other groups at the start of treatment). *p < 0.05 and **p < 0.01 in indicated comparisons; ns, not significant. The orange line indicates a calculated additive model, with 95% confidence intervals indicated as a yellow shaded range. *p* value of synergy of DT2219 + ABT199 over additivity: 0.020. **d** Immunoblot analysis of Bcl-xL, Bcl-2, and Mcl-1 in the spleen (*n* = 3 mice in each group). Proteins were extracted from the spleens of mice after euthanization when they reached the experimental endpoint as described in the “Materials and methods” section. **e**–**g** Quantification of Bcl-xL, Bcl-2, and Mcl-1 levels in **d**. The data presented are mean ± SEM (*n* = 3 mice per each group). a, b, and c, *p* < 0.05 vs. VEH, DT2216, and ABT199, respectively. **h** Body weight changes over time in DFTL-28776 PDX cell-bearing mice after DT2216 and/or ABT199 treatment as shown in Fig. 5. The data presented are mean ± SEM (*n* = 5 for VEH and *n* = 4 for other groups at the start of treatment)

We injected DFTL-28776 PDX cells via the tail vein of NOD/SCID mice, which results in orthotopic engraftment in the bone marrow, spleen, liver, and blood [8, 31]. Mice with > 1% engraftment of DFTL-28776 PDX cells in blood were randomly assigned to four different treatment groups (Supplementary Fig. S3). They received treatment with vehicle, DT2216, ABT199, or DT2216 plus ABT199 (Fig. 4a). ABT199 had minimal effects on blood T-PLL burden and modestly increased the median survival (17 days vs. 26 days in vehicle-treated mice); DT2216 was slightly more effective than ABT199 in inhibiting DFTL-28776 PDX cell blood engraftment and prolonging survival (Fig. 4b, c and Supplementary Fig. S3). In contrast, the combination of DT2216 and ABT199 substantially inhibited the increase of DFTL-28776 PDX cells in blood and markedly extended the median survival time of the mice compared to all other treatments (Fig. 4b, c and Supplementary Fig. S3). More importantly, the survival analysis of the mice using the Bliss independence model reveals that DT2216 plus ABT199 achieved synergy based on a significantly superior survival (*p* = .020) compared with that expected from additivity. These effects of DT2216 were associated with a significant reduction in Bcl-xL expression in spleen DFTL-28776 PDX cells (Fig. 4d, e). Of note, ABT199 treatment led to Mcl-1 upregulation in spleen T-PLL cells, which was blocked by the addition of DT2216 (Fig. 4d, g). None of the treatments affected the body weight of the mice (Fig. 4h).

**Fig. 5:**
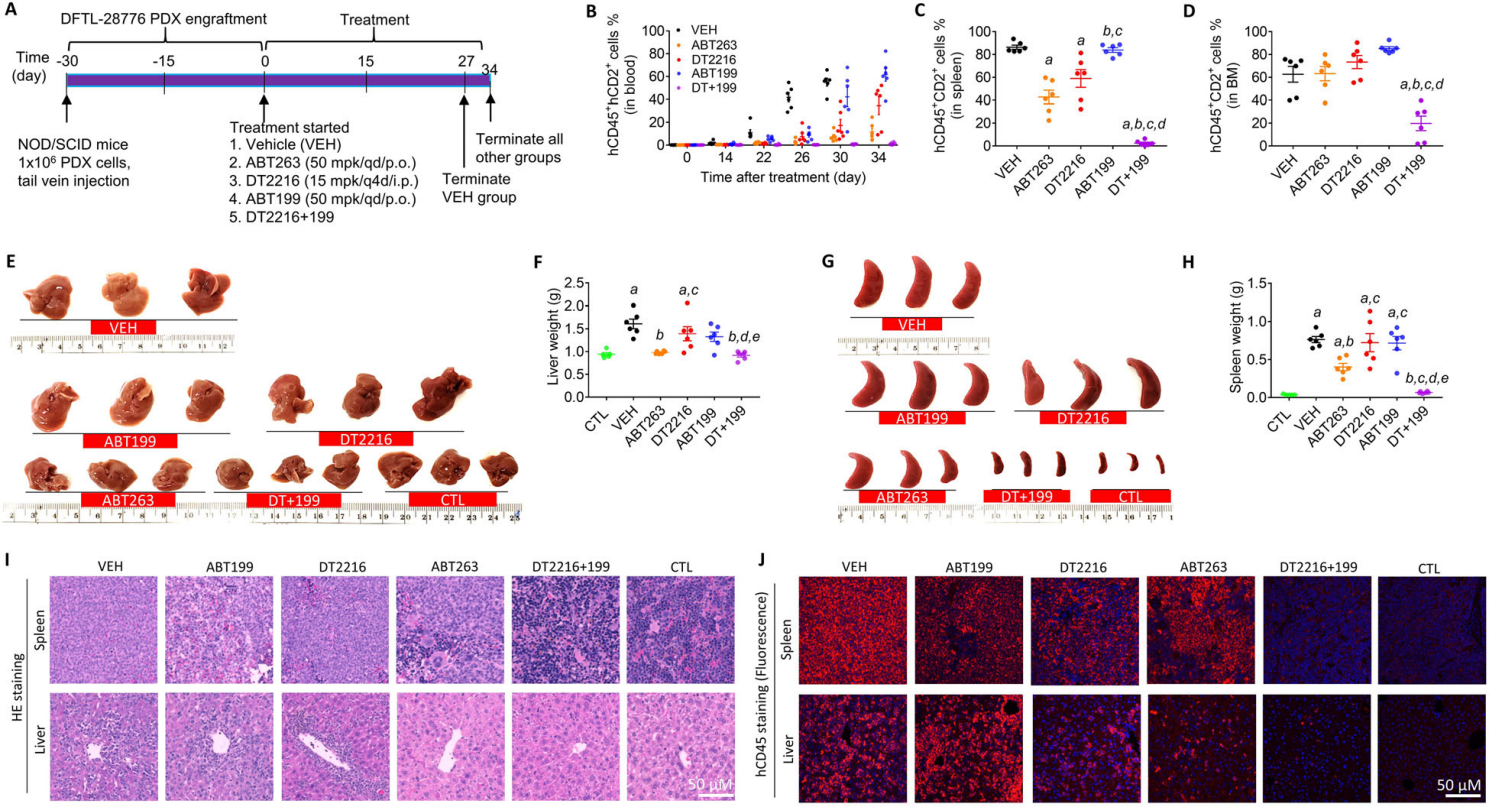
DT2216 more effectively reduces TCL burdens of TCL PDX mice when combined with ABT199*.***a** Illustration of the experimental design of the second study on DFTL-28776 PDX xenograft model. A group of age-matched female NOD/SCID control (CTL) mice without DFTL-28776 engraftment or any other treatments were included for comparative purposes. **b** Changes in blood DFTL-28776 PDX cell burden over time after the treatment as shown in **a**. The data presented are mean ± SEM (*n* = 6 mice per each group). **c** Spleen DFTL-28776 PDX cell burden in the PDX xenograft mice after treatment as shown in **a**. The data presented are mean ± SEM (*n* = 6 mice per each group). a, b, c, and d, *p* < 0.05 vs. VEH, ABT263, DT2216, and ABT199, respectively. **d** Bone marrow DFTL-28776 PDX cell burden in the DFTL-28776 PDX xenograft mice after treatment as shown in **a**. The data presented are mean ± SEM (*n* = 6 mice per each group). a, b, c, and d, *p* < 0.05 vs. VEH, ABT263, DT2216, and ABT199, respectively. **e** Liver images of DFTL-28776 PDX cell-engrafted mice. **f** Liver weight in DFTL-28776 PDX xenograft mice after treatment as shown in **a**. The data presented are mean ± SEM (*n* = 5 for the CTL group and *n* = 6 mice for other groups). a, b, c, d, and e, *p* < 0.05 vs. CTL, VEH, ABT263, DT2216, and ABT199, respectively. **g** Spleen images of DFTL-28776 PDX cell engrafted mice. **h** Spleen weight in DFTL-28776 PDX xenograft mice after treatment as shown in **a**. The data presented are mean ± SEM (*n* = 5 for the CTL group and *n* = 6 mice for other groups). a, b, c, d, and e, *p* < 0.05 vs. CTL, VEH, ABT263, DT2216, and ABT199, respectively. **i** Hematoxylin & eosin (HE) staining of the spleens and livers from DFTL-28776 PDX cell-engrafted mice. **j** Human CD45 (hCD45) staining of the spleens and livers from DFTL-28776 PDX cell-engrafted mice. Red color, hCD45 staining with vectastain; blue color, nuclear staining with DAPI

To better characterize the effects of ABT199 and/or DT2216 on DFTL-28776 PDX, we repeated the study presented in Fig. 4a but initiated the treatments when the mice had a mean blood DFTL-28776 PDX cell engraftment of around 0.1%. The vehicle-treated mice were humanely euthanized 27 days after the initiation of the treatments when the mice became nearly moribund due to a high burden of the disease (Fig. 5a and Supplementary Fig. S4). The remaining groups of mice were terminated 7 days later. This experiment allowed us to analyze the effects of different treatments on not only blood engraftment of DFTL-28776 PDX cells, but also the engraftment of DFTL-28776 PDX cells in the spleen, liver, and bone marrow. In addition, we added the ABT263 treatment group into the study to compare the effects of Bcl-xL inhibition vs. degradation on DFTL-28776 PDX. The results from this study confirmed that the combination of DT2216 and ABT199 was more effective than either of these agents alone in suppression of DFTL-28776 PDX cell blood engraftment as shown in the previous study (Figs. 4b and 5b). ABT263 was also more effective than ABT199 or DT2216, but less effective than DT2216 plus ABT199, in inhibition of DFTL-28776 PDX cell blood engraftment (Fig. 5b and Supplementary Fig. S4). ABT199 had no significant effect on the size/weight of the spleen and liver and DFTL-28776 PDX cell engraftment in the spleen and bone marrow, whereas DT2216 only moderately reduced DFTL-28776 PDX cell engraftment in the spleen but had no significant effect on all other parameters measured (Fig. 5b–j). In contrast, DT2216 plus ABT199 normalized the size/weight of the spleen and liver and substantially reduced DFTL-28776 PDX cell engraftment in the spleen and bone marrow. ABT263 also normalized the size/weight of the liver but was less effective in suppression of DFTL-28776 PDX cell engraftment in the spleen and had no effect on DFTL-28776 PDX cell engraftment in the bone marrow (Fig. 5b–j). More importantly, the combination treatment with DT2216 and ABT199 did not result in further reduction in blood platelets and white blood cells (WBCs) than the treatment with either agent alone (Supplementary Fig. S4B, C). In fact, the DFTL-28776 PDX mice treated with DT2216 plus ABT199 exhibited less reduction in blood platelets and WBCs than the mice treated with ABT199. This effect may be attributed to a better control of TCL by the combination treatment, which likely further lessens the bone marrow suppression caused by TCL bone marrow infiltration to promote the production of platelets and WBCs. Collectively, these results demonstrate that the combination of DT2216 and ABT199 is an effective treatment for TCLs that rely on both Bcl-xL and Bcl-2 for survival. The synergy between DT2216 and ABT199 is potentially greater than dual inhibition of Bcl-xL and Bcl-2 by ABT263, which also induces dose-limiting thrombocytopenia in humans.

## Discussion

Our findings demonstrate that targeting Bcl-xL using DT2216 can selectively kill Bcl-xL-dependent TCL cells without causing significant platelet toxicity. Moreover, the combination of DT2216 with a Bcl-2 inhibitor can also effectively target TCLs that depend on both Bcl-xL and Bcl-2 for survival. The ability to combine DT2216 with ABT199 in vivo suggests that DT2216 could be used in many different combinations to synergistically increase TCL killing without inducing thrombocytopenia, which cannot be achieved by the use of either Bcl-xl-specific inhibitors such as A-1155463 or the Bcl-xL/2-dual inhibitor ABT263 because Bcl-xL is essential for platelet survival and these inhibitors causes severe thrombocytopenia [33]. The improved potency of DT2216 against TCLs compared to Bcl-xL inhibitors is likely attributed to its PROTAC property. As conventional Bcl-xL inhibitors, ABT263 or A-1155463 requires constant occupancy of the BH3-binding sites to sequester Bim and other proapoptotic Bcl-2 family proteins [34]. The effectiveness of ABT263 or A-1155463 in vivo against TCLs depends on the maintenance of long-term and high-level drug exposure, which is difficult to achieve without causing significant toxicity [9, 12]. In contrast, DT2216 acts as a Bcl-xL PROTAC that can catalytically induce Bcl-xL degradation in a sub-stoichiometric manner in TCL cells but not in platelets because VHL is abundantly expressed in TCL cells but is poorly expressed in platelets [13]. As such, the effect of DT2216 is not limited by equilibrium occupancy and does not cause thrombocytopenia. In addition, the catalytic nature of DT2216 as a PROTAC results in an extended duration of action beyond the plasma pharmacokinetic half-life. Therefore, its therapeutic effect is long-lasting and requires only once or twice a week dosing to be effective in vivo [13], which makes DT2216 not only therapeutically more effective, but also less toxic than ABT263 or other Bcl-xL-specific inhibitors. Furthermore, after ABT263 is converted into DT2216, the PROTAC molecule becomes a specific Bcl-xL degrader without inducing Bcl-2 degradation. Similar phenomena have been reported previously [35, 36]. The lack of efficient degradation of Bcl-2 by DT2216 may be attributed to the significant reduction of its binding affinity to Bcl-2 [14], lack of the lysine for ubiquitination, and inability of DT2216 to form ternary complex with VHL and Bcl-2 in cells [13]. Since Bcl-2 inhibition with ABT-263 can lead to neutropenia and lymphocytopenia [37], the inability of DT2216 to induce Bcl-2 degradation may confer to DT2216 additional advantages by reducing the on-target toxicity of ABT263 on Bcl-2.

As expected, single-agent DT2216 was unable to cure mice engrafted with TCLs. The mechanisms by which TCL cells evade DT2216 (or combination with ABT199) have yet to be elucidated. One possibility is that that specific microenvironments can serve as sanctuaries. This could involve the suppression of pro-apoptotic factors through various soluble factors, cell adhesion molecules, and extracellular matrix molecules. In fact, previous studies have implicated a range of factors, including CXCL12/CXCR4, NOTCH ligands and receptors, VCAM-1/VLA-4 and 5, fibronectin/VLA-4 and 5, hyaluronate and collagen/CD44 [38, 39]. Alternatively, TCL cells may upregulate other Bcl-2 anti-apoptotic proteins to compensate for the loss of Bcl-xL, as observed in patients who developed adaptive resistance after long-term responses to venetoclax [40, 41]. Further studies are needed to clarify the mechanisms, first in preclinical models and then in humans, in order to design rational combinations that obviate the development of resistance to DT2216.

## Conclusions

In summary, DT2216 is highly active both in vitro and in vivo against TCL cells with dependence on Bcl-xL and does not cause significant thrombocytopenia. Combinations that target additional dependences do not increase the toxicity of DT2216, suggesting that it could enhance the efficacy of a broad range of therapeutics.

## Supplementary information

**Additional file 1: Supplementary Figure 1.** Effect of DT2216 on *BCL2L1*, *BCL2*, and *MCL1* mRNA expressions in TCL cells. **Supplementary Figure 2.** Effect of DT2216 on MJ, MAC2A and L82 TCL cells. **Supplementary Figure 3.** Effect of DT2216 and/or ABT199 on TCL PDX cell blood engraftment and body weight in TCL PDX mice. **Supplementary Figure 4**. The combination therapy of DT2216 and ABT199 is more effective than either agent alone or ABT263 against TCL PDX in mice. **Supplementary Table 1.** Effects of chemotherapy drugs, Bcl-2 family protein inhibitors and DT2216 on DFTL-28776 TCL PDX cells in vitro.

## Data Availability

All data and materials supporting the conclusion of this study have been included within the article and the supplemental data.

## Abbreviations

ANOVA: Analysis of variance
CBC: Complete blood count
cCaspase-3: Cleaved caspase-3
CI: Combination index
cPARP: Cleaved ADP ribose polymerase
CTCL: Cutaneous T cell lymphoma
EC_50_: Half maximal effective concentration
HRP: Horse radish peroxidase
PDX: Patient-derived xenograft
PARP: ADP ribose polymerase
PGE_1_: Prostaglandin E1
PROTAC: Proteolysis targeting chimera
PRP: Platelet-rich plasma
PTCL: Peripheral T cell lymphoma
qPCR: Quantitative polymerase chain reaction
TCL: T cell lymphoma
QVD: QVD-OPh
T-PLL: T cell prolymphocytic leukemia
VHL: Von Hippel-Lindau
SCLC: Small cell lung cancer
T-ALL: T cell acute lymphoblastic leukemia
WBCs: White blood cells
