# Supplementary material for: DT2216—a Bcl-xL-specific degrader is highly active against Bcl-xL-dependent T cell lymphomas
Source: J Hematol Oncol. 2020 Jul 16;13:95. doi: 10.1186/s13045-020-00928-9 (PMC7364785; doi:10.1186/s13045-020-00928-9)
Supplement: Supplementary file 1 — Additional file 1: Supplementary Figure 1. Effect of DT2216 on BCL2L1, BCL2, and MCL1 mRNA expressions in TCL cells. Supplementary Figure 2. Effect of DT2216 on MJ, MAC2A and L82 TCL cells. Supplementary Figure 3. Effect of DT2216 and/or ABT199 on TCL PDX cell blood engraftment and body weight in TCL PDX mice. Supplementary Figure 4. The combination therapy of DT2216 and ABT199 is more effective than either agent alone or ABT263 against TCL PDX in mice. Supplementary Table 1. Effects of chemotherapy drugs, Bcl-2 family protein inhibitors and DT2216 on DFTL-28776 TCL PDX cells in vitro. [file 13045_2020_928_MOESM1_ESM.docx]

**Supplementary Information**

He Y *et al*. DT2216 – a Bcl-xL specific degrader is highly active against Bcl-xL-dependent T-cell lymphomas

| **Supplementary Item** | **Title** |
| --- | --- |
| Supplementary Figure 1 | Effect of DT2216 on *BCL2L1*, *BCL2*, and *MCL1* mRNA expressions in TCL cells |
| Supplementary Figure 2 | Effect of DT2216 on MJ, MAC2A and L82 TCL cells |
| Supplementary Figure 3 | Effect of DT2216 and/or ABT199 on TCL PDX cell blood engraftment and body weight in TCL PDX mice |
| Supplementary Figure 4 | The combination therapy of DT2216 and ABT199 is more effective than either agent alone or ABT263 against TCL PDX in mice |
| Supplementary Table 1 | Effects of chemotherapy drugs, Bcl-2 family protein inhibitors and DT2216 on DFTL-28776 TCL PDX cells in vitro |

**
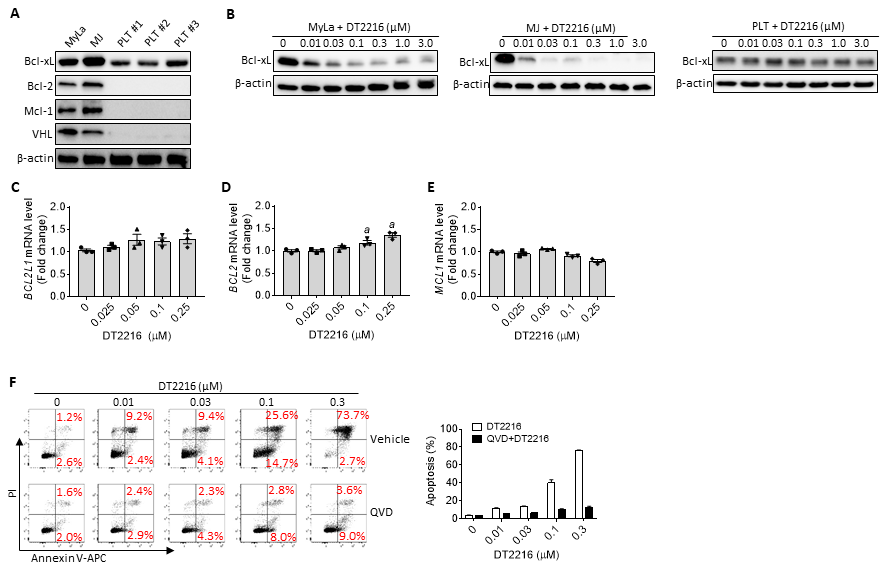
**

**Supplementary Figure S1.** (A) A represent immunoblot analysis of Bcl-xL, Bcl-2, Mcl-1 and VHL expression in MyLa and MJ cells, and PLTs. (B) A represent immunoblot analysis of Bcl-xL expression in MyLa and MJ cells, and PLTs after they were incubated with increasing concentrations of DT2216 for 16 h. (C-E) Effect of DT2216 on *BCL2L1*, *BCL2*, and *MCL1* mRNA expressions in TCL cells. The levels of *BCL2L1* (C), *BCL2* (E) and *MCL1* (F) mRNA in MyLa cells were measured by qPCR after treatment with indicated concentrations of DT2216 for 16 h. The data are presented as mean ± SD (*n=*3 replicates). (F) DT2216 induced apoptosis in MyLa cells in a dose-dependent manner, which was blocked by pretreatment with 10 μM QVD. MyLa cells were pretreated with or without 10 μM QVD for 4 h, and then treated with indicated concentrations of DT2216 for 24 h. Representative flow cytometric analyses of apoptosis are shown in the left panel. PI, propidium iodide. The data presented in the right panel are mean ± SD (*n=*3 replicates).


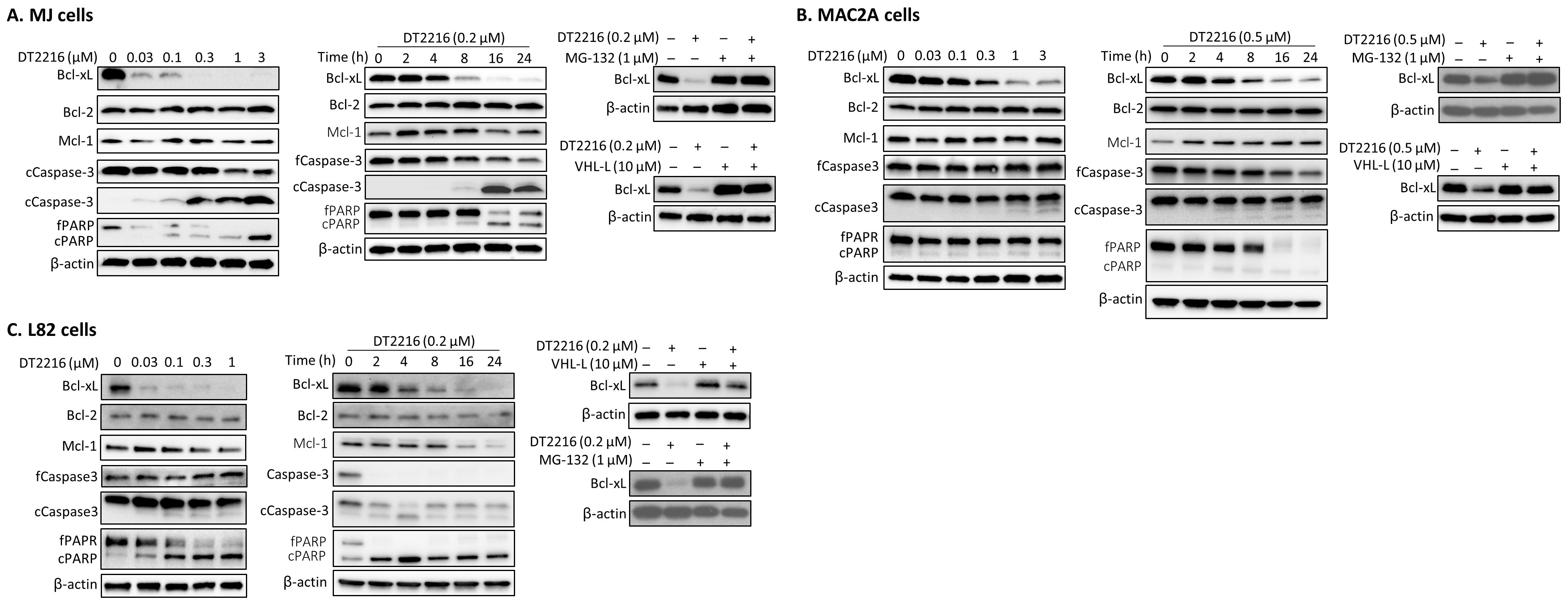


**Supplementary Figure S2. Effect of DT2216 on MJ, MAC2A, and L82 TCL cells.** (A) DT2216 induced degradation of Bcl-xL and cleavage of caspase-3 and PARP in MJ cells in a dose- and time-dependent manner. Pretreatment of MJ cells with the proteasome inhibitor MG-132 or VHL ligand (VHL-L) blocked the degradation of Bcl-xL by DT2216 (right panel). (B) DT2216 induced degradation of Bcl-xL and cleavage of caspase-3 and PARP in MAC2A cells in a dose- and time-dependent manner. Pretreatment of MAC2A cells with MG-132 or VHL-L blocked the degradation of Bcl-xL by DT2216 (right panel). (C) DT2216 induced degradation of Bcl-xL and cleavage of caspase-3 and PARP in L82 cells in a dose- and time-dependent manner. Pretreatment of L82 cells with MG-132 or VHL-L blocked the degradation of Bcl-xL by DT2216 (right panel). For right panels of (A) to (C), cells were pretreated with 1 μM MG-132 or 10 μM VHL-L for 2 h, and treated with DT2216 for 16 h.


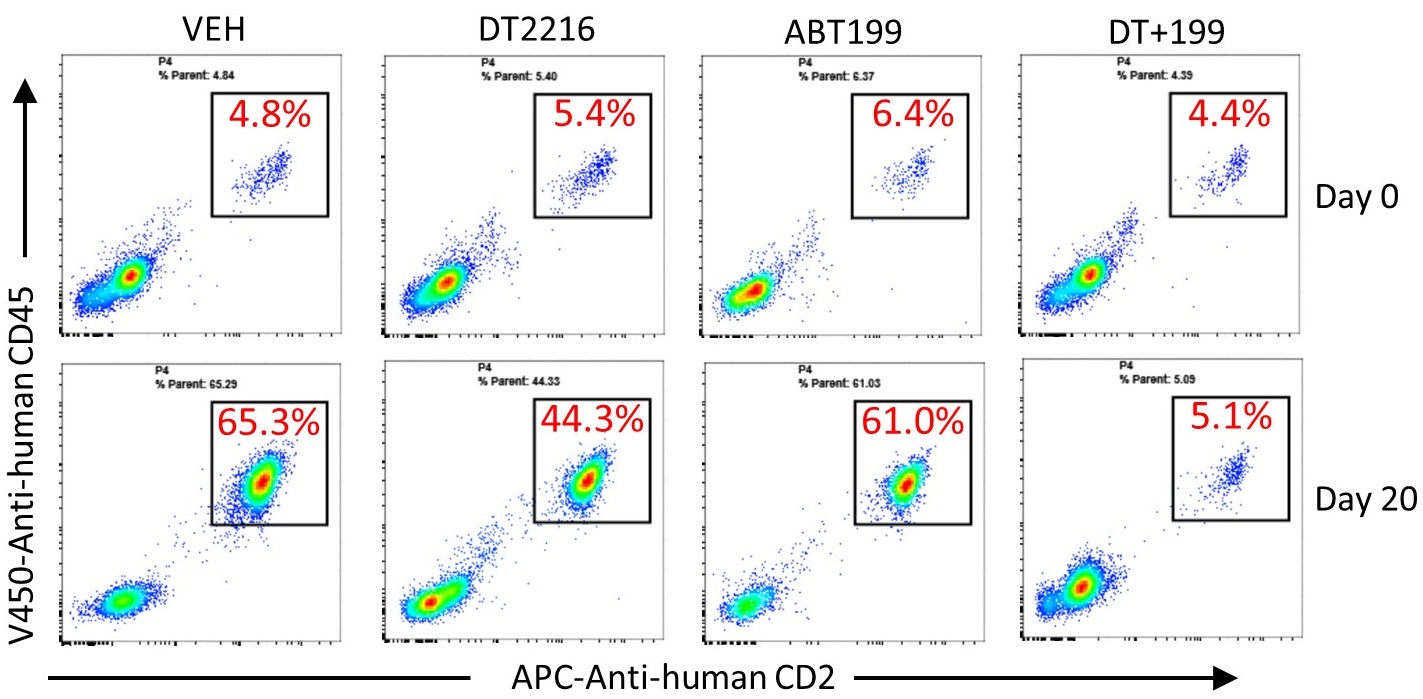


**Supplementary Figure S3.** **Effect of DT2216 and/or ABT199 on TCL PDX cell blood engraftment in TCL PDX mice.** Representative flow cytometric analyses of DFTL-28776 PDX cell engraftment in blood of PDX mice before (day 0) and after (day 20) being treated with DT2216 and/or ABT199 as shown in Figure 5A.


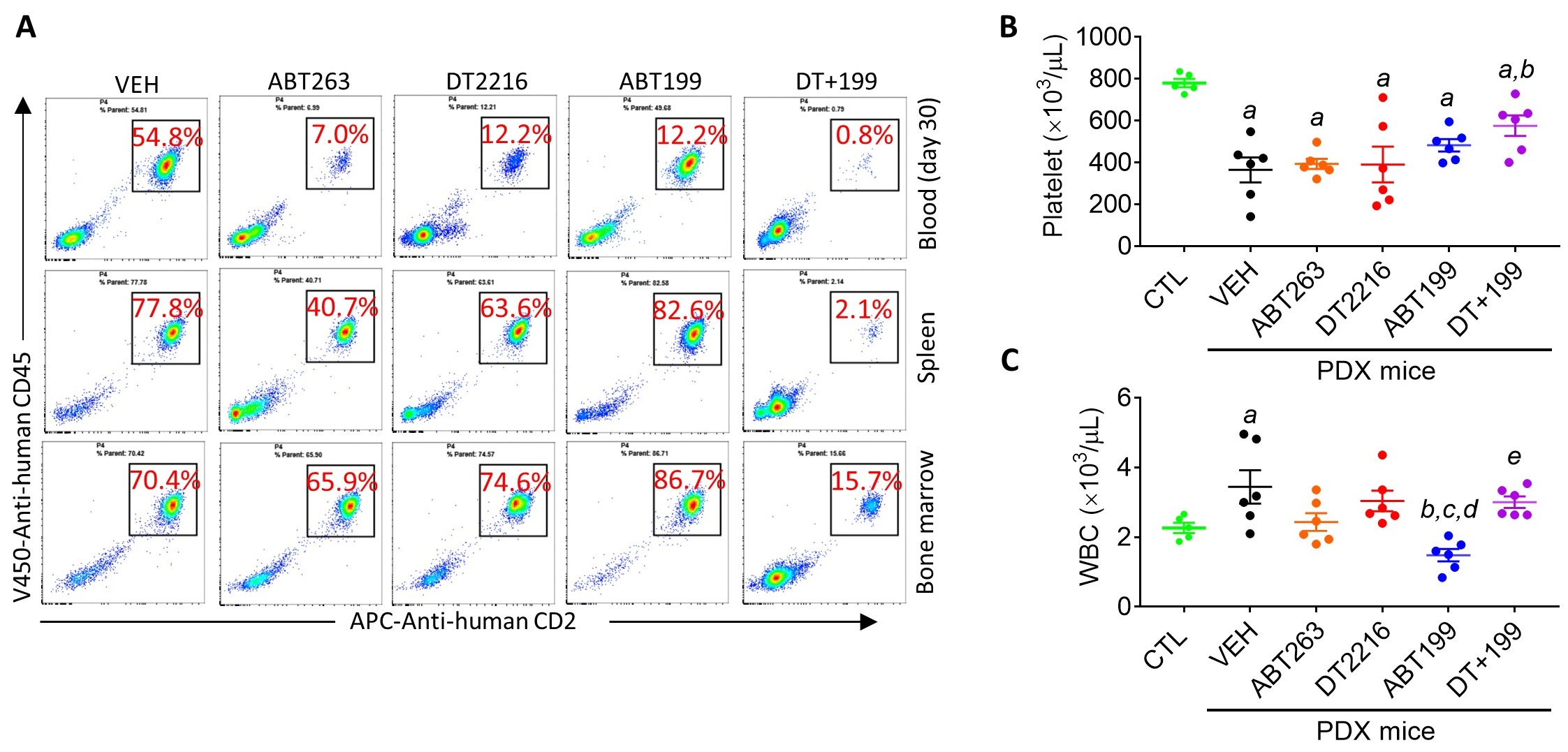


**Supplementary Figure S4. Effect of ABT263, DT2216 and/or ABT199 on TCL PDX cell blood engraftment, and on platelet and white blood cell (WBC) counts in TCL PDX mice.** (A) Representative flow cytometric analyses of DFTL-28776 PDX cell engraftment in the spleen, bone marrow and blood. Blood platelet (B) and WBC (C) counts in DFTL-28776 PDX xenograft mice on day 26 after treatment and in control mice (CTL) without DFTL-28776 PDX xenograft. The data presented are mean ± SEM (*n=*5 for the control group and *n=*6 mice for other groups). *a, b*, *c, d* and *e*, *p*< 0.05 *vs*. CTL, VEH, ABT263, DT2216 and ABT199, respectively.

| **Supplementary Table 1. Effects of chemotherapy drugs, Bcl-2 family protein inhibitors and DT2216 on DFTL-28776 PDX cells *in vitro*** | | | |
| --- | --- | --- | --- |
| Compounds | EC_25_ (µM) | EC_50_ (µM) | EC_75_ (µM) |
| Doxorubicin | 0.054 | 0.330 | 3.861 |
| Etoposide | 0.546 | >10 | 22.702 |
| Vincristine | 0.031 | 0.230 | 3.727 |
| A-1155463 | 0.003 | 0.600 | 23.236 |
| ABT199 | 0.010 | 0.190 | 4.447 |
| S63845 | 0.002 | 0.060 | 1.746 |
| ABT263 | 0.023 | 0.060 | 0.342 |
| DT2216 | 0.047 | 0.480 | 5.088 |
| DT2216+ABT199 | 0.006 | 0.040 | 1.199 |

EC_25_, EC_50_, and EC_75_: the concentration that led to 25%, 50% and 75% maximal effectiveness, respectively.
